# Learning from online complaints mentioning nurses in Türkiye: a topic modelling study

**DOI:** 10.1186/s12913-026-15254-y

**Published:** 2026-07-30

**Authors:** Mehmet Gülşen, Ayşe Karadaş

**Affiliations:** 1 Department of Medical Services and Techniques, İvrindi Vocational School of Health Services, Balıkesir University, Balıkesir, Türkiye; 2 Department of Nursing, Faculty of Health Sciences, Balıkesir University, Balıkesir, Türkiye

**Keywords:** Patient complaints, Healthcare delivery, Nurses, Patient experience, Topic modelling, Online complaints, Quality improvement, Türkiye

## Abstract

**Background:**

Online complaint platforms provide unsolicited accounts of patient and family experiences and can offer valuable insights into healthcare delivery. However, little is known about what complaints mentioning nurses reveal about care processes in Türkiye. This study identified the main themes in online patient and family complaints that explicitly mentioned nurses and examined how these complaints reflected nursing, medical, and system-level aspects of healthcare delivery.

**Methods:**

We conducted a descriptive, exploratory study of 11,674 publicly available complaint narratives posted on a major online complaint platform in Türkiye between September 2022 and February 2025. Complaints explicitly referring to nurses were identified using a Turkish-language keyword meaning “nurse”. After text cleaning, lemmatisation, tokenisation, and stop-word removal, topic modelling was performed. Model selection was guided by coherence and interpretability. Descriptive statistics were used to summarise corpus characteristics and topic prevalence. The final model identified 13 topics, which were subsequently grouped by the researchers for interpretive purposes as nursing-centred, medical-centred, or system-centred.

**Results:**

The 13 topics indicated that complaints mentioning nurses were not limited to direct nursing care but reflected interconnected nursing, medical, and system-level problems. Medical-centred topics accounted for the largest share of complaints (43.56%), followed by nursing-centred topics (36.40%) and system-centred topics (20.04%). The most prevalent topics concerned examination, diagnosis, and referral problems (24.27%); ward environment, responsiveness, and nursing care (17.79%); and emergency department access and waiting times (13.51%). Overall, complaints mentioning nurses most often reflected problems in clinical communication, responsiveness, procedural care, access, waiting times, and care coordination.

**Conclusions:**

Within this keyword-defined corpus, online complaints mentioning nurses offer insight into perceived shortcomings in healthcare provision. Although many complaints concerned nursing-sensitive issues such as communication, responsiveness, and procedural care, a substantial proportion reflected wider medical and organisational problems. Monitoring such complaints may support quality improvement across multidisciplinary care pathways.

**Clinical trial number:**

Not applicable.

**Supplementary Information:**

The online version contains supplementary material available at https://doi.org/10.1186/s12913-026-15254-y.

## Background

Patient complaints are increasingly recognised as a valuable source of evidence for improving healthcare quality and safety [1]. They can reveal problems in clinical care, communication, access, and organisational processes that may not be fully captured by conventional monitoring systems [2]. Formal complaints also provide insight into how patients and families experience negative encounters with healthcare services [3]. Nurse-related complaints are particularly important because nurses occupy a highly visible position in frontline care and are closely involved in communication, coordination, and day-to-day patient management [4]. Analysing complaints mentioning nurses may therefore provide insight not only into nursing-sensitive concerns, but also into broader issues that patients and families encounter across service pathways.

The expansion of public digital complaint platforms has increased the availability of unsolicited patient and family narratives about healthcare services. Unlike structured surveys, these narratives are produced in service users’ own words and can capture dissatisfaction across the care pathway, including interpersonal conduct, delays, coordination failures, and environmental conditions [5]. In Türkiye, previous studies have examined online or institutional complaint data in hospitals and health services, showing that patient complaints frequently concern communication problems, waiting times, and organisational shortcomings [6, 7]. However, this literature has generally focused on healthcare organisations more broadly rather than on complaints mentioning nurses specifically.

The scale and unstructured nature of complaint text make manual analysis difficult when large datasets are involved. Natural language processing offers a practical means of transforming large volumes of free-text feedback into interpretable patterns relevant to service improvement [8]. Topic modelling has been used to analyse patient experience narratives and other forms of healthcare feedback across various settings [9, 10]. It has also been applied to nursing-related text corpora, suggesting its usefulness for examining large-scale narrative data in health services research [11, 12].

Despite these developments, little is known about what publicly posted complaints mentioning nurses reveal about healthcare delivery in Türkiye. This is an important gap because references to nurses in complaint narratives may indicate direct problems in nursing care, but they may also reflect wider perceived problems in diagnosis, referral, waiting-time management, coordination, and administrative processes. Accordingly, this study aimed to identify the main themes in online patient and family complaints that explicitly mentioned nurses in Türkiye and to examine how these themes were distributed across nursing-centred, medical-centred, and system-centred aspects of healthcare delivery.

## Methods

### Study design

This study employed a descriptive and exploratory design based on topic modelling to examine publicly available online complaint narratives mentioning nurses in Türkiye. Latent Dirichlet Allocation (LDA), a widely used probabilistic topic modelling method, was applied to identify latent themes within unstructured textual data [13]. Data analysis was conducted in Python (version 3.11.0) on a Windows 10 operating system, using scientific computing libraries commonly applied in data-intensive research [14]. The analytical workflow used pandas 2.3.3 for data handling, NLTK 3.9.2 for stop-word processing, JPype 1.6.0 to run Zemberek-NLP locally via the zemberek-full.jar distribution (version 0.17.1), Gensim 4.4.0 for corpus construction and LDA estimation, and Matplotlib for visualisation. NumPy 2.3.5, SciPy 1.16.3, and scikit-learn 1.8.0 were used for supplementary validation analyses. Tokens that could not be processed by the morphological analyser were retained in lowercase form to prevent preprocessing exceptions from removing complaint content. The core Python workflow and the specifications used for corpus preprocessing, LDA estimation, dominant-topic assignment, and temporal topic summarisation are provided as Supplementary File S2.

The study proceeded in four stages: data collection, text preprocessing, topic modelling, and interpretation of results.

### Data source and data collection

Complaint data were obtained from Şikayetvar (sikayetvar.com), a public online platform on which users post complaints about services, institutions, and consumer experiences. To construct a corpus focused on complaints mentioning nurses, relevant records were identified using the Turkish keyword “hemşire” (“nurse”). This strategy was intended to capture narratives that explicitly referred to nurses as opposed to general complaints about healthcare services.

The dataset was stored in Excel format and imported into Python for preprocessing and modelling. A total of 11,674 complaint narratives were retrieved together with associated metadata, including submission date, complaint text, and view count. The corpus covered complaints posted between September 2022 and February 2025, and data extraction was completed on 7 April 2025.

### Text preprocessing

Before topic modelling, the raw texts underwent a structured preprocessing pipeline designed to improve data quality and model performance, consistent with recommendations for applied topic modelling in communication and health research [15]. First, all complaint records were imported into a pandas DataFrame, and date variables were converted into Python datetime objects. A year_month variable was subsequently derived to support temporal analysis.

Each complaint text was then normalised by converting all characters to lowercase and removing punctuation and numeric characters using regular expressions. Because the corpus consisted of Turkish-language narratives, morphological normalisation was performed using the Zemberek Turkish morphological analyser via JPype. For each token, the best disambiguated morphological analysis was obtained, and the first lemma was retained, thereby reducing inflected forms to a common lexical base. The lemmatised text was then tokenised by whitespace.

To reduce noise, tokens shorter than three characters were discarded, and non-informative terms were removed using a combined stop-word list consisting of NLTK’s Turkish stop words and a curated set of domain-specific stop words. The domain-specific list was developed to remove high-frequency conversational, deictic, and generic complaint expressions that occurred widely in the corpus but did not contribute substantively to topic differentiation. This step was intended to reduce lexical noise while preserving terms with clinical, organisational, procedural, or interpersonal relevance. After preprocessing, the analytical corpus comprised 11,674 complaint narratives and 1,031,684 tokens, with a mean processed document length of 88.37 tokens. The output of this stage was a cleaned token list per complaint for use in dictionary construction and LDA estimation.

### Topic modelling and interpretation

Following preprocessing, the tokenised complaints were converted into a Gensim dictionary and a bag-of-words corpus. To reduce sparsity and remove both overly rare and overly common terms, the dictionary was filtered using filter_extremes(no_below = 10, no_above = 0.5), thereby excluding terms that appeared in fewer than 10 documents or in more than 50% of documents. These thresholds were chosen to preserve sufficient lexical variation while improving topic stability.

Model selection was based on statistical diagnostics and substantive interpretability across alternative topic solutions. LDA models with k = 10–15 topics were estimated using five random seeds for each topic number. Model performance was evaluated using training and held-out c_v coherence, held-out perplexity, topic diversity, and stability across random initialisations. Topic diversity was calculated as the proportion of unique terms among the top 10 words across all topics. Topic-word stability was assessed using matched Jaccard similarity of top-word sets, and document-level assignment stability was examined using the adjusted Rand index. The 13-topic solution was selected because it achieved the highest mean c_v coherence in both the training and held-out corpora and provided the most substantively interpretable topic structure.

The final LDA model was estimated with 13 topics (num_topics = 13), 10 passes (passes = 10), automatic alpha optimisation (alpha = ‘auto’), a fixed random seed (random_state = 42), and per-word topic assignments enabled (per_word_topics = True). Ten passes allowed sufficient iteration over the corpus while keeping the model configuration reproducible. Automatic alpha optimisation was used to allow variation in document–topic concentration across complaint narratives; the learned alpha values ranged from 0.095 to 0.524, with a mean of 0.221. The topic–word prior eta was retained at the default symmetric Gensim setting and was not separately optimised; therefore, no learned eta values were estimated. The selected model achieved a c_v coherence score of 0.572.

Because algorithmic metrics alone cannot fully capture semantic nuance, topic interpretation also included manual review of topic keywords, topic-word distributions, and representative complaint narratives [16]. Topic labels were not derived from high-probability terms in isolation. A provisional label was retained only when it captured a coherent semantic pattern across the topic keywords, the broader topic-word distribution, and the reviewed complaint narratives, while remaining distinguishable from other topics. Alternative interpretations were discarded when they depended on isolated keywords, lacked consistent support in the reviewed narratives, or overlapped substantially with another topic. The semantic validity of the dominant-topic assignments was assessed through manual review. For each of the 13 topics, 10 complaints assigned to that topic as the dominant topic were randomly selected, yielding a validation sample of 130 complaints. This topic-stratified design ensured that all topics, including those with relatively low prevalence, were examined with equal scrutiny. The sample was intended as a focused semantic audit of the topic assignments rather than as a basis for estimating corpus-level frequencies. Two researchers independently reviewed each complaint and its assigned topic label to determine whether the label reflected the main content of the complaint. Any differences in judgement were resolved through discussion. In addition, the topic labels, dominant keywords, and representative complaint excerpts were reviewed by three healthcare professionals with expertise in nursing and health services research to assess their clinical and organisational plausibility. This step supported the interpretation of whether the model-derived topic assignments and labels were meaningful at the document and domain levels. As a comparative check, BERTopic was explored during model development because alternative topic modelling approaches may yield substantively different outputs [17]. For this exploratory comparison, BERTopic was implemented using the configuration reported in Supplementary File S2, including the embedding model, UMAP settings, HDBSCAN settings, and the constrained 13-topic configuration used for comparison with LDA. The density-based BERTopic solutions produced overly coarse structures with only two substantive topic clusters despite c_v coherence values of 0.534 and 0.608. Conversely, the constrained 13-topic BERTopic solution produced a more fragmented structure and yielded lower c_v coherence than the retained LDA model (0.499 vs. 0.572). LDA was therefore retained as the primary analytical approach.

For prevalence reporting, each complaint was assigned to the topic with the highest posterior probability. This dominant-topic assignment was used only as a descriptive summary of the main thematic signal in each complaint, since LDA represents documents as mixtures of topics and individual complaints may contain multiple concerns. Two researchers independently classified the 13 LDA-derived topics into three domains: nursing-centred, medical-centred, and system-centred. Inter-rater agreement was complete (100% observed concordance; Cohen’s κ = 1.00). The κ value was calculated from the initial independent classification of the 13 aggregate topics; because all classifications were identical, no disagreement resolution or iterative refinement of the codebook was required. This classification was applied post hoc to support health services interpretation rather than being treated as a direct output of the model. The domain criteria and topic assignment rationale are provided in Supplementary File S1.

### Ethical considerations

This study was conducted in accordance with the ethical principles of the Declaration of Helsinki. The study analysed publicly available, user-generated online complaint narratives and did not involve direct interaction with human participants. The complaint texts were obtained from Şikayetvar, an open online platform on which users voluntarily post service-related experiences. Permission to use the data for research purposes was obtained from Şikayetvar. Human ethics approval and consent to participate were not applicable because the study used publicly accessible textual data retrospectively, did not collect identifiable private information, and reported only aggregated findings and anonymised excerpts. Representative complaint excerpts were shortened and anonymised; names, institutional identifiers, locations, and other potentially identifiable contextual details were removed. No personally identifiable information was reported in the manuscript. Although the complaint narratives were publicly accessible, they were treated as potentially sensitive digital trace data; therefore, all reporting was limited to aggregated findings or anonymised excerpts with identifiable contextual details removed.

## Results

### Descriptive characteristics of the complaint dataset

The dataset comprised 11,674 unique complaint narratives retrieved from a public online platform, covering the period from September 2022 to February 2025. All complaints explicitly referred to nurses.

The corpus contained 1,335,256 words in total. Complaint length ranged from 12 to 663 words, with a mean of 114.38 words per complaint. After preprocessing, document length averaged 88.37 tokens (SD = 58.22; median = 71; range = 10–479). The vocabulary comprised 74,766 unique processed terms before dictionary filtering, corresponding to a type–token ratio of 0.072. The cumulative view count exceeded 44 million, indicating substantial public visibility of the complaints analysed. View counts were right-skewed, with a median of 2,606 views (IQR = 1,410–4,636; 90th percentile = 8,080). Complaints in the highest decile of view count were more concentrated in examination, diagnosis, and referral problems (36.0% vs. 24.3% overall), physician communication and consultation problems (15.7% vs. 12.9%), and maternity and newborn care processes (11.2% vs. 6.4%). Descriptive statistics are presented in Table 1.

**Table 1:** Descriptive characteristics of the complaint corpus

Characteristic	Value
Number of complaint narratives	11,674
Study period	September 2022–February 2025
Total word count	1,335,256
Mean words per complaint	114.38
Word count range per complaint	12–663
Total view count	44,203,869

### Topic modelling results

The topic modelling analysis identified 13 distinct topics within the complaint corpus. Model diagnostic analyses across k = 10–15 and five random seeds supported this solution, as the 13-topic model produced the highest mean c_v coherence in both the training corpus (mean = 0.471, SD = 0.015) and the held-out corpus (mean = 0.456, SD = 0.025). Topic diversity for the 13-topic solution was 0.674, while topic-word stability showed modest consistency across random initialisations (mean matched Jaccard similarity = 0.315). Document-level assignment stability, assessed using the adjusted Rand index, was 0.153. These stability values indicated modest reproducibility across model initialisations rather than strong topic stability. Although the 10-topic model produced the lowest held-out perplexity, the 13-topic solution offered the best overall balance across coherence, topic diversity, and interpretability and was therefore retained. All complaints mentioned nurses, yet many narratives also reflected broader clinical and organisational processes. For interpretive clarity, the researchers subsequently grouped the 13 model-derived topics according to their dominant focus as nursing-centred, medical-centred, or system-centred. Medical-centred topics accounted for the largest share of complaints (43.56%), followed by nursing-centred topics (36.40%) and system-centred topics (20.04%).

The most prevalent individual topics were examination, diagnosis, and referral problems (24.27%), ward environment, responsiveness, and nursing care (17.79%), and emergency department access and waiting times (13.51%). Together, these three topics accounted for more than half of all complaints.

Other notable themes covered both clinical and organisational aspects of care. These included physician communication and consultation problems (12.93%), blood sampling and intravenous procedures (7.28%), maternity and newborn care processes (6.36%), women’s health service experiences (4.45%), intensive care communication and family experience (3.71%), surgical scheduling and access delays (3.26%), paediatric venous access and blood sampling (3.16%), public health service and governance concerns (1.93%), appointment, payment, and administrative problems (0.70%), and postoperative stay and night-time care concerns (0.64%). A summary of topics, keywords, and prevalence is presented in Table 2.

**Table 2:** Topics identified through Latent Dirichlet Allocation and their prevalence

Topic	Topic label	Dominant focus	Top keywords	*n*	%	Representative complaint excerpts
1	Paediatric venous access and blood sampling	Nursing-centred	vein; child; intravenous line; daughter; fever; infant; needle; paediatric; blood draw	369	3.16	“The nurse struggled to insert an IV line in my feverish child, and my child was left frightened and crying.” “The nurse inserted the needle into my five-month-old baby’s arm […] and moved it around to find the vein.”
2	Surgical scheduling and access delays	System-centred	surgery; request; hospital; application; process; result; scheduling; date; follow-up	381	3.26	“The nurse said the surgery process would be followed up, but our operation date kept being postponed.” “We were called back for surgery, but the nurses said they had not been informed […] and we were left waiting outside the ward.”
3	Maternity and newborn care processes	Medical-centred	birth; baby; wife; hospital; admission; mother; woman; caesarean; waiting; doctor	742	6.36	“After the birth, no nurse came to check my baby or inform us about the ward process.” “When I told the nurse that my baby had stopped feeding, she dismissed my concern. […] A lactation consultant later noticed the breathing problem.”
4	Emergency department access and waiting times	System-centred	patient; waiting time; emergency; request; delay; ambulance; admission; unattended care	1,577	13.51	“We waited for hours in the emergency department, but the nurse did not intervene or provide any information.” “Despite severe breathing difficulty, the triage nurse assigned my mother to the green area […] with 95 patients ahead of us.”
5	Public health service and governance concerns	System-centred	health; family health; public health; ministry; complaint; vaccine; service; institution; regulation	225	1.93	“The public health nurse called us for vaccination, but no nurse was there when we arrived.” “Our family health nurse would not administer my baby’s tuberculosis vaccine. […] Another centre also refused because the child was not registered there.”
6	Physician communication and consultation problems	Medical-centred	doctor; examination; waiting time; question; explanation; repeated consultation	1,510	12.93	“I asked a question about the prescription but could not obtain an explanation.” “When I tried to ask about my test results, the doctor dismissed my questions […] and told me to ask them at the next consultation.”
7	Examination, diagnosis, and referral problems	Medical-centred	doctor; examination; waiting time; emergency; diagnosis; incorrect diagnosis; medication; information; referral	2,833	24.27	“I was diagnosed without a proper examination, and the nurse did not answer my question.” “When I asked the nurse about the treatment, I learned that the doctor had not entered the medication into the system […] and the nursing staff had also overlooked it.”
8	Ward environment, responsiveness, and nursing care	Nursing-centred	patient; hospital; cleanliness; environment; attention; behaviour; care; neglect	2,077	17.79	“We called the nurses many times, but they did not come to the patient’s room.” “When we asked the nurses for help, they told us to ask the cleaning staff instead.”
9	Blood sampling and intravenous procedures	Nursing-centred	blood; serum; vein; needle; intravenous line; blood draw; repeated attempts; test; result	850	7.28	“During blood sampling, the nurse repeatedly inserted the needle and caused unnecessary pain.” “The nurse could not establish intravenous access and called another nurse while the needle was still in my arm. […] My pain was ignored.”
10	Appointment, payment, and administrative problems	System-centred	appointment; information; payment; doctor; device; time; fee; system; delay; cancellation	82	0.70	“Although we had an appointment, several patients were called at the same time, and the nurse provided no clear information.” “The appointment and payment were completed online, but I was told to return in person […] to obtain a refund for the excess charge.”
11	Intensive care communication and family experience	Nursing-centred	intensive care; mother; father; heart; admission; companion; information; patient	433	3.71	“My relative said the intensive care nurse shouted at him during the night.” “Doctors and nurses gave us conflicting information about the treatment […] and for two days we were not allowed to see my father in intensive care.”
12	Women’s health service experiences	Nursing-centred	woman; birth; hospital; public hospital; behaviour; doctor; midwife; ward	520	4.45	“After birth, we asked for breastfeeding support but received a rude response.” “The midwives and nurses examined patients with the door open […] without protecting their privacy.”
13	Postoperative stay and night-time care concerns	System-centred	food; night; surgery; room; stay; meal; patient; morning; information; care	75	0.64	“After surgery, the nurse call button did not work at night, and we could not reach a nurse.” “When my dressing came off during the night, the nurse said she did not know how to replace it […] and told me to wait until morning.”

Note. Percentages represent dominant-topic prevalence based on the topic assigned as dominant for each complaint narrative. Domain classification was conducted independently by two researchers using a topic classification codebook and showed complete agreement (Cohen’s κ = 1.00). Percentages may not sum to exactly 100% because of rounding

### Nursing-centred topics

Nursing-centred topics accounted for over one-third of all complaints and primarily reflected issues related to direct care, patient–nurse interactions, and the care environment. The most prominent theme in this category was ward environment, responsiveness, and nursing care (17.79%), which included dissatisfaction with attentiveness, response to patient needs, perceived quality of care, and ward conditions.

Technical and procedural aspects of nursing practice were also evident. Complaints relating to blood sampling and intravenous procedures (7.28%) and paediatric venous access and blood sampling (3.16%) frequently described repeated attempts at venous access and associated distress. Intensive care communication and family experience (3.71%) highlighted communication and interaction problems in high-acuity settings, while women’s health service experiences (4.45%) reflected concerns about care processes and professional interactions.

### Medical- and system-centred topics

Medical- and system-centred topics captured complaints primarily related to diagnostic processes, physician interactions, waiting times, access to services, and organisational procedures. Although these topics were not nursing-specific in their primary focus, they were included because all complaints mentioned nurses.

Medical-centred topics were dominated by examination, diagnosis, and referral problems (24.27%) and physician communication and consultation problems (12.93%), often reflecting delays, perceived diagnostic errors, repeated consultations, or insufficient information. System-centred topics included emergency department access and waiting times (13.51%), surgical scheduling and access delays (3.26%), public health service and governance concerns (1.93%), appointment, payment, and administrative problems (0.70%), and postoperative stay and night-time care concerns (0.64%). Together, these topics pointed to concerns related to access, coordination, administrative processes, and inpatient service conditions.

Across these categories, complaints frequently linked nurses to broader care processes, suggesting that narratives mentioning nurses were often embedded within interconnected clinical and organisational contexts.

**Fig. 1:**
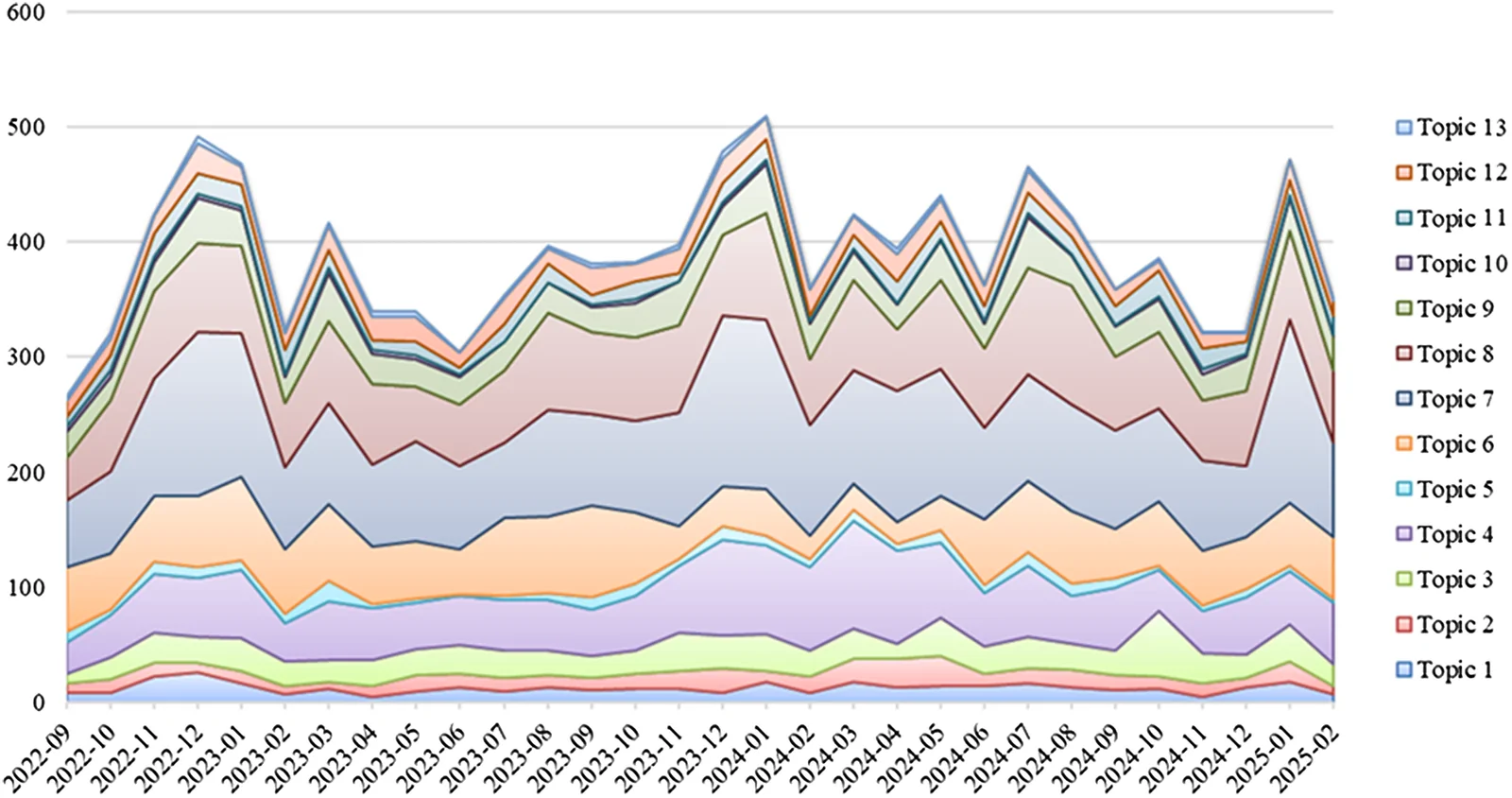
Monthly distribution of topics identified through Latent Dirichlet Allocation

### Temporal distribution of topics

The monthly distribution of complaints remained broadly stable over the study period, with periodic increases observed in November 2022, January 2023, November 2023, and January 2025 (Fig. 1). The most prevalent topics remained consistently prominent over time. The temporal visualisation was used descriptively to contextualise complaint patterns over the study period rather than to test formal seasonal or longitudinal trends.

**Fig. 2:**
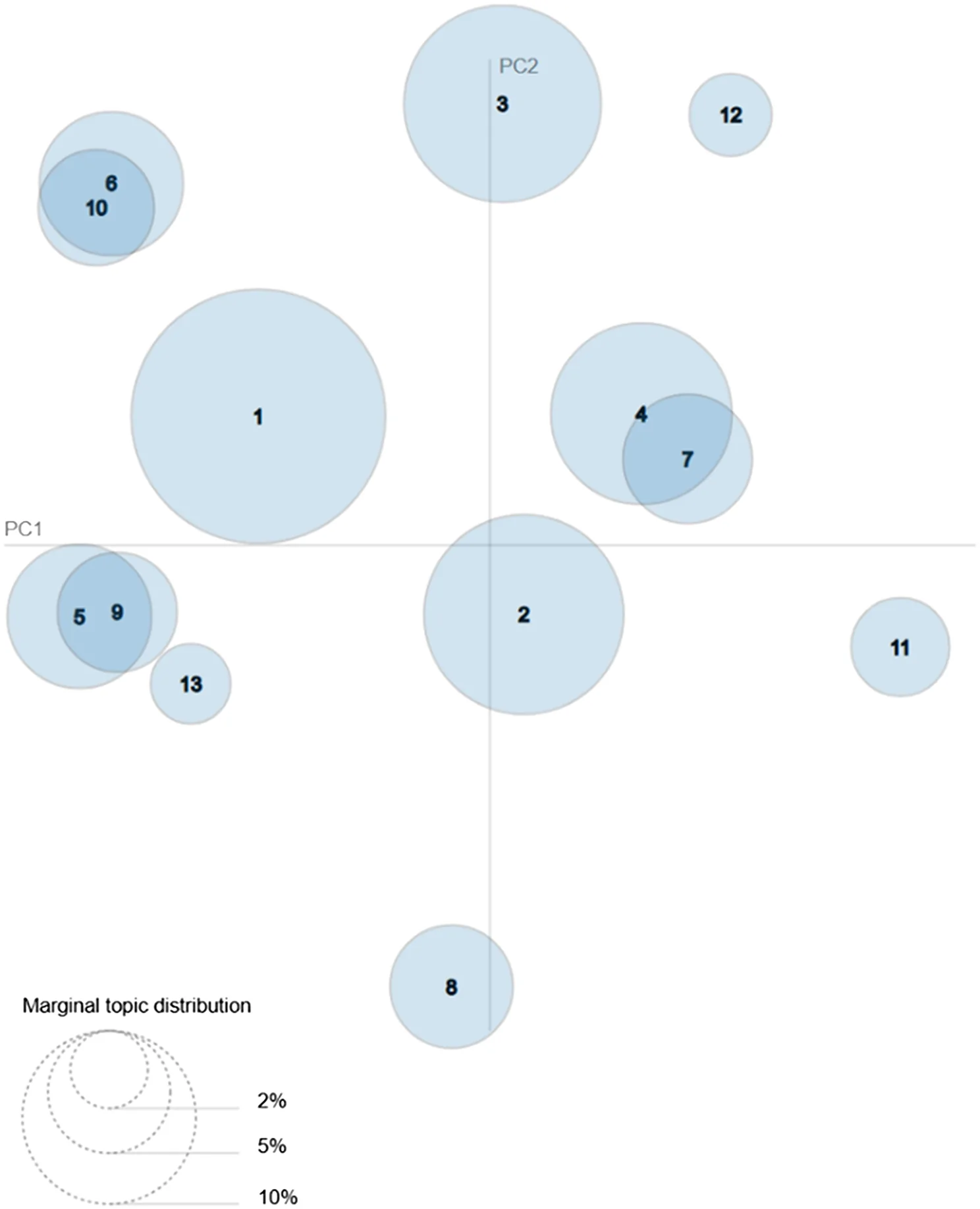
Intertopic distance map (via multidimensional scaling). Note. Circle sizes represent the marginal topic distribution (mean topic proportions across documents), whereas Table 2 reports dominant-topic prevalence

### Intertopic relationships

The intertopic distance map indicated that most topics were relatively distinct, with limited overlap between a small number of topic pairs (Fig. 2). This overlap suggests partial thematic convergence, particularly where clinical and organisational issues intersect.

**Fig. 3:**
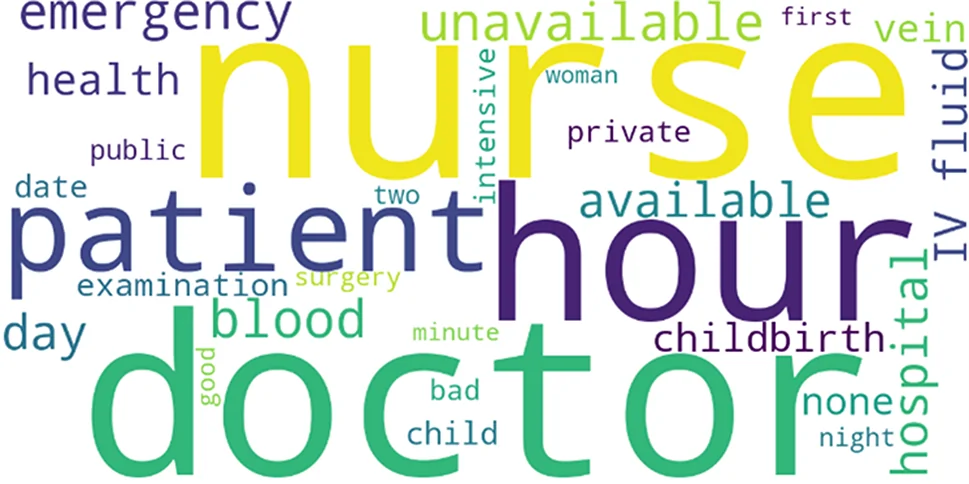
Word cloud

### Word frequency patterns

The most frequently occurring words in the corpus included nurse, doctor, patient, hour, blood, and emergency (Fig. 3). The co-occurrence of professional roles and time-related terms suggests that complaints mentioning nurses were often situated within broader clinical and organisational processes.

## Discussion

Online complaints explicitly mentioning nurses in this study reflected interconnected nursing-sensitive, medical, and system-level problems rather than issues confined to nursing practice alone. This pattern is consistent with complaint research showing that patient complaints often span clinical, management, and relationship domains [1, 2]. It also aligns with evidence from Türkiye. Çınar et al. [6] reported that online complaints about hospitals involved management, relationship, and clinical problems, including delays in access, examination and diagnosis problems, ward care, and disrespect. Karaduman et al. [18] similarly found that poor staff attitudes and long waiting times were among the most frequent reasons for formal complaints. Building on this literature, the present study shows that complaints mentioning nurses may provide a visible entry point into perceived gaps in care provision, including communication, responsiveness, diagnostic and referral processes, waiting times, and administrative coordination.

A key finding was that nursing-centred complaints were dominated by ward environment, responsiveness, and nursing care. This broadly aligns with Street et al. [4], whose analysis of complaints concerning nursing and midwifery care found that almost half of the coded complaint categories fell within the Relationship domain. Their study also showed that complaints categorised as relating to nursing or midwifery often involved other healthcare professionals [4]. In the present study, complaints mentioning nurses were likewise not limited to isolated interpersonal incidents but were often embedded in broader frontline care conditions. This interpretation is also compatible with Eriksen et al. [3], who identified recurrent negative experiences relating to access, information, inappropriate care, and trust in providers. Turkish complaint studies similarly suggest that communication problems, staff attitudes, waiting times, and organisational shortcomings are recurring features of patient dissatisfaction [6, 7].

Complaints related to blood sampling and intravenous procedures and to paediatric venous access and blood sampling frequently described repeated attempts, distress, and insufficient explanation. These findings are in line with Cooke et al. [19], who reported that peripheral intravenous cannulation can be painful, stressful, and frustrating for consumers, and that first-attempt failure was more common in children (64%) than in adults (40%). Similar themes emerged in Piazza et al.’s [20] study of paediatric blood draws, which highlighted the importance of communication, comfort measures, and rapport in shaping the procedural experience. Taken together, these studies support the interpretation that technical performance and procedural communication are closely intertwined in how patients and families evaluate routine invasive care.

The topic labelled “intensive care communication and family experience” similarly highlights the importance of dialogue and emotional support in high-acuity settings. Al-Shamaly [21] identified communication patterns, barriers, and the importance of addressing psychological and emotional needs in intensive care, while Leong et al. [22] showed that intensive care patients and family members expressed needs for information, reassurance, and supportive care. The present findings are consistent with this literature and suggest that online complaints may help reveal how unmet informational and emotional needs surface as patient dissatisfaction.

A particularly important result was that medical-centred topics accounted for the largest share of complaints. Topics such as “examination, diagnosis, and referral problems” and “physician communication and consultation problems” were more prevalent than any single nursing-centred category. This suggests that many complaints mentioning nurses were thematically aligned with medical or organisational processes rather than with nursing care alone. One possible explanation is that patients and relatives may perceive nurses as the most accessible representatives of the healthcare team, even when the underlying problem concerns diagnosis, referral, medical decision-making, or service coordination. In this sense, complaints mentioning nurses may reflect both role ambiguity in patient perceptions and perceived difficulties in interprofessional coordination across care pathways. The system-centred topics reinforce this interpretation. Complaints about emergency department access and waiting times, surgical scheduling and access delays, and appointment, payment, and administrative problems indicate that many complaints mentioning nurses were thematically associated with delays, coordination difficulties, and barriers to access. In emergency care, patients who reported not waiting for triage had higher triage and nursing satisfaction scores than those who reported waiting [23]. Nationwide data likewise suggest that waiting time and length of stay are relevant to patient satisfaction [24]. Collectively, these studies indicate that delays, interpersonal conduct, and service flow are recurrent features of how patients evaluate healthcare delivery. In the Turkish context, these patterns may be particularly visible in online complaints because patients and relatives often encounter nurses at multiple transition points, including triage, ward admission, procedural care, discharge preparation, and information seeking.

Overall, these findings suggest that complaints mentioning nurses should be interpreted as a composite signal of multiple challenges within healthcare services. Some clearly concern nursing-sensitive issues, especially communication, responsiveness, interpersonal conduct, and routine procedures. Others, however, reflect wider clinical and organisational processes in which nurses are highly visible but not necessarily the primary source of the problem. This distinction matters because a superficial reading of complaint data may attribute wider coordination, access, or service-flow problems too narrowly to individual professional performance. From a health services perspective, complaint data can instead offer valuable insight into how patients and families encounter the healthcare system in practice [2].

### Limitations

This study has several limitations. First, the data were drawn from a single online complaint platform and reflect the perspectives of individuals who chose to post complaints publicly. The findings therefore cannot be assumed to represent the full range of patient experiences in Türkiye. Moreover, because corpus eligibility required explicit use of the Turkish keyword “hemşire” (“nurse”), complaints concerning healthcare delivery that did not contain this term were outside the sampling frame. The topic prevalence estimates therefore describe this keyword-defined subset of online complaints and should not be interpreted as the distribution of concerns across healthcare complaints in Türkiye more generally. Online complaints are likely to reflect the experiences of patients or relatives who have internet access, are familiar with public complaint platforms, and are willing to share negative healthcare experiences publicly. Patients who prefer formal hospital complaint channels, who do not use digital platforms, or who choose not to complain publicly may therefore be under-represented. Such platforms may also over-represent sudden, serious, or emotionally distressing incidents, while longer-term, less visible, or silently endured dissatisfaction may be less likely to appear. Second, the analysis relied on textual complaint data and was not triangulated with survey, interview, or administrative data. Because demographic information was not systematically available, differences by age, gender, region, or hospital type could not be examined. Future studies could extend the descriptive temporal analysis through formal longitudinal testing and incorporate sentiment and subgroup analyses where suitable metadata are available. The accuracy of individual complaints could not be independently verified, and duplicate or repeated postings could not be fully excluded. The analysis was also limited to Turkish-language complaints and to the defined study period. Third, some contextual nuance may have been lost during preprocessing. Despite modest stability, the combined statistical and semantic validation supported retaining the 13-topic solution as an interpretable rather than definitive representation of the corpus. Finally, the three-domain classification was used to organise the LDA-derived topics for health services interpretation. Although this classification showed complete inter-rater agreement, some complaints may still have reflected overlapping nursing, medical, and organisational processes. Because topic prevalence was reported using dominant-topic assignment, the percentages should be interpreted as descriptive indicators of the main thematic signal in each complaint rather than as mutually exclusive representations of all concerns contained in the narratives.

### Implications for practice

The findings of this study suggest several practical implications. Complaints related to responsiveness, attentiveness, and ward conditions may indicate a need to review ward-level care processes, workload distribution, and mechanisms for responding to patient and family needs. For hospital administrators, recurring complaint topics may function as early warning signals for service areas requiring targeted quality improvement, particularly ward responsiveness, emergency department flow, procedural care, and communication during diagnostic or referral processes. Complaints about repeated blood sampling and intravenous access attempts, particularly in children, point to the potential value of strengthening procedural training and establishing clearer escalation processes when venous access is difficult. Communication problems were another recurring concern, suggesting that structured information-sharing practices and regular family updates may help improve patient and family experience, especially in high-acuity settings such as intensive care. Complaint monitoring could also be incorporated into routine quality improvement cycles by periodically reviewing topic patterns, assigning recurrent themes to relevant clinical or administrative units, and tracking whether corrective actions reduce similar complaints over time. Importantly, many complaints mentioning nurses reflected wider service-level concerns, including waiting times, diagnostic and referral delays, and weak care coordination. Complaints mentioning nurses may therefore be useful not only as feedback on nursing practice but also as a signal for multidisciplinary quality improvement.

## Conclusions

Within this keyword-defined corpus, online complaints mentioning nurses in Türkiye should not be interpreted as complaints about nursing care alone, but rather as mixed narratives that may reflect nursing-sensitive concerns, medical processes, and broader organisational challenges encountered through frontline healthcare interactions. Although many complaints reflected nursing-sensitive issues such as responsiveness, communication, and routine procedures, a larger combined share was thematically associated with diagnostic, referral, waiting-time, and administrative problems in which nurses were nevertheless explicitly mentioned. Analysed carefully, such complaints may provide useful feedback on recurrently reported concerns in care pathways and inform quality improvement across healthcare services. These findings reflect complaints posted on a single online platform and should not be generalised to all patients, healthcare institutions, or the wider healthcare system in Türkiye.

## Supplementary Information

Below is the link to the electronic supplementary material.

Supplementary Material 1

Supplementary Material 2

## Data Availability

The datasets used and analysed during this study are available from the corresponding author on reasonable request.

## Abbreviations

LDA: Latent dirichlet allocation
