# Supplementary material for: Learning from online complaints mentioning nurses in Türkiye: a topic modelling study
Source: BMC Health Serv Res. 2026 Jul 30;26:1497. doi: 10.1186/s12913-026-15254-y (PMC13726020; doi:10.1186/s12913-026-15254-y)
Supplement: Supplementary file 1 [file 12913_2026_15254_MOESM1_ESM.docx]

**Supplementary File S1. Topic classification codebook and domain assignment criteria**

## Table S1.1. Domain classification criteria

| **Domain** | **Classification criterion** |
| --- | --- |
| Nursing-centred | Topics primarily referring to direct nursing care, nurse–patient or nurse–family interaction, ward responsiveness, procedural nursing care, information provision involving nurses, or care environment issues in which nursing practice was the dominant focus. |
| Medical-centred | Topics primarily referring to diagnosis, examination, clinical decision-making, medical treatment, referral, physician consultation, maternity or newborn clinical processes, or other medical processes in which the dominant issue was clinical assessment or treatment planning rather than nursing care alone. |
| System-centred | Topics primarily referring to access, waiting times, scheduling, appointment or payment processes, emergency department flow, administrative coordination, governance, inpatient service conditions, or organisational processes that shaped the care experience. |

## Table S1.2. Topic classification codebook

| **Topic** | **Final label** | **Domain** | **Brief rationale** |
| --- | --- | --- | --- |
| 1 | Paediatric venous access and blood sampling | Nursing-centred | Classified as nursing-centred because the topic focused on venous access, blood sampling, repeated attempts, and child distress during procedures usually performed or coordinated by nurses. |
| 2 | Surgical scheduling and access delays | System-centred | Classified as system-centred because the dominant issue concerned scheduling, access, follow-up, and delays in the surgical pathway rather than direct care by a single professional group. |
| 3 | Maternity and newborn care processes | Medical-centred | Classified as medical-centred because the topic centred on birth, caesarean section, newborn and maternal clinical processes, admission, and treatment-related care pathways. |
| 4 | Emergency department access and waiting times | System-centred | Classified as system-centred because the topic primarily reflected emergency access, waiting times, patient flow, delays, ambulance-related issues, and unattended care within emergency service organisation. |
| 5 | Public health service and governance concerns | System-centred | Classified as system-centred because the topic referred to vaccination, family health services, ministry or institutional processes, regulation, and public health governance. |
| 6 | Physician communication and consultation problems | Medical-centred | Classified as medical-centred because the topic focused on physician consultation, examination, explanation, questions about treatment, and repeated clinical encounters. |
| 7 | Examination, diagnosis, and referral problems | Medical-centred | Classified as medical-centred because the dominant issue concerned diagnosis, referral, medication, examination quality, information provision related to clinical decision-making, and perceived diagnostic problems. |
| 8 | Ward environment, responsiveness, and nursing care | Nursing-centred | Classified as nursing-centred because the topic referred to ward care, response to calls, cleanliness, attention to patients, behaviour, neglect, and perceived quality of day-to-day nursing care. |
| 9 | Blood sampling and intravenous procedures | Nursing-centred | Classified as nursing-centred because the topic centred on blood draw, serum administration, intravenous lines, repeated needle attempts, and procedural care. |
| 10 | Appointment, payment, and administrative problems | System-centred | Classified as system-centred because the topic was dominated by appointment scheduling, payment, fees, cancellations, device/system issues, and administrative delays. |
| 11 | Intensive care communication and family experience | Nursing-centred | Classified as nursing-centred because the topic focused on information provision, interaction with relatives, companion experience, and perceived conduct of intensive care staff, particularly nurses. |
| 12 | Women’s health service experiences | Nursing-centred | Classified as nursing-centred because the topic concerned women’s health service encounters, nurse or midwife behaviour, ward support, breastfeeding-related support, and interpersonal care experiences. |
| 13 | Postoperative stay and nighttime care concerns | System-centred | Classified as system-centred because the topic referred to inpatient stay, nighttime service conditions, meals, room conditions, call-button access, and organisational arrangements after surgery. |
