# Supplementary material for: Learning from online complaints mentioning nurses in Türkiye: a topic modelling study
Source: BMC Health Serv Res. 2026 Jul 30;26:1497. doi: 10.1186/s12913-026-15254-y (PMC13726020; doi:10.1186/s12913-026-15254-y)
Supplement: Supplementary file 2 [file 12913_2026_15254_MOESM2_ESM.docx]

**Supplementary File S2. Python workflow for corpus preprocessing, LDA estimation, and topic assignment**

This script reproduces the core analytical workflow used for preprocessing the complaint corpus, estimating the LDA topic model, assigning dominant topics to complaint narratives, and summarising monthly topic distributions.

import os

import re

from pathlib import Path

import pandas as pd

import matplotlib.pyplot as plt

import nltk

import jpype

import jpype.imports

from jpype.types import *

from gensim import corpora, models

from gensim.models import CoherenceModel

from nltk.corpus import stopwords

\# ============================================================

\# 1. User-defined paths and model settings

\# ============================================================

BASE_DIR = Path(".").resolve()

\# Adapt these paths to the local working directory

DATA_PATH = BASE_DIR / "data" / "Hem.xlsx"

ZEMBEREK_DIR = BASE_DIR / "zemberek"

OUTPUT_DIR = BASE_DIR / "outputs"

OUTPUT_DIR.mkdir(parents=True, exist_ok=True)

NUM_TOPICS = 13

RANDOM_STATE = 42

PASSES = 10

NO_BELOW = 10

NO_ABOVE = 0.5

\# ============================================================

\# 2. Start Zemberek

\# ============================================================

jar_files = [

os.path.join(ZEMBEREK_DIR, file)

for file in os.listdir(ZEMBEREK_DIR)

if file.endswith(".jar")

]

if len(jar_files) == 0:

raise FileNotFoundError(

"No .jar file was found in the Zemberek directory. "

"Please check the ZEMBEREK_DIR path."

)

classpath = ";".join(jar_files)

if not jpype.isJVMStarted():

jpype.startJVM(classpath=classpath)

TurkishMorphology = jpype.JClass("zemberek.morphology.TurkishMorphology")

morphology = TurkishMorphology.createWithDefaults()

\# ============================================================

\# 3. Zemberek-based lexical normalisation

\# ============================================================

def zemberek_stem(text):

"""

Applies Zemberek-based lexical normalisation where possible.

If Zemberek analysis fails for a token, the lowercased token is retained.

"""

cleaned = []

for word in text.split():

try:

analysis = morphology.analyze(word)

results = morphology.disambiguate(word, analysis).bestAnalysis()

if results.size() > 0:

lemma = results.get(0).getLemmas()[0]

cleaned.append(str(lemma).lower())

else:

cleaned.append(word.lower())

except Exception:

cleaned.append(word.lower())

return " ".join(cleaned)

\# ============================================================

\# 4. Stop-word list

\# ============================================================

nltk.download("stopwords")

stop_words = set(stopwords.words("turkish"))

domain_stopwords = [

"ben", "bana", "beni", "benim", "biz", "bizim", "sen", "sana", "seni",

"siz", "sizin", "o", "onun", "ki", "de", "da", "şu", "bu", "dedi",

"dedim", "diyerek", "şöyle", "böyle", "şekilde", "sonra", "önce",

"kadar", "hala", "artık", "zaten", "gibi", "ile", "ise", "veya",

"ya", "hem", "bile", "çok", "az", "fazla", "oldu", "oldum",

"olduğum", "yaptım", "ettim", "gel", "geldi", "gitti", "gittik",

"geldik", "gidince", "yani", "daha", "ne", "nasıl", "hiç", "hep",

"ama", "fakat", "çünkü", "eğer", "diye", "bir", "her", "biraz",

"başka"

]

stop_words.update(domain_stopwords)

\# ============================================================

\# 5. Text preprocessing

\# ============================================================

def preprocess_text(text):

"""

Lowercases text, removes numbers and punctuation, applies lexical

normalisation, tokenises by whitespace, removes stop words, and discards

tokens shorter than three characters.

"""

text = str(text).lower()

text = re.sub(r"\d+", "", text)

text = re.sub(r"[^\w\s]", "", text)

text = zemberek_stem(text)

tokens = text.split()

tokens = [

word for word in tokens

if word not in stop_words and len(word) > 2

]

return tokens

\# ============================================================

\# 6. Data import and preprocessing

\# ============================================================

df = pd.read_excel(DATA_PATH)

required_columns = {"time", "text"}

missing_columns = required_columns.difference(df.columns)

if missing_columns:

raise ValueError(f"Missing required columns: {missing_columns}")

df["time"] = pd.to_datetime(df["time"], errors="coerce")

df["year_month"] = df["time"].dt.to_period("M").astype(str)

df["tokens"] = df["text"].apply(preprocess_text)

df = df[df["tokens"].map(len) > 0].copy()

df["raw_word_count"] = df["text"].astype(str).str.split().map(len)

df["processed_token_count"] = df["tokens"].map(len)

print("Number of retained complaint narratives:", len(df))

print("Mean raw word count:", round(df["raw_word_count"].mean(), 2))

print("Mean processed token count:", round(df["processed_token_count"].mean(), 2))

\# ============================================================

\# 7. Dictionary and corpus construction

\# ============================================================

texts = df["tokens"].tolist()

dictionary = corpora.Dictionary(texts)

print("Vocabulary size before filtering:", len(dictionary))

dictionary.filter_extremes(

no_below=NO_BELOW,

no_above=NO_ABOVE

)

print("Vocabulary size after filtering:", len(dictionary))

corpus = [dictionary.doc2bow(text) for text in texts]

\# ============================================================

\# 8. LDA model estimation

\# ============================================================

lda_model = models.LdaModel(

corpus=corpus,

id2word=dictionary,

num_topics=NUM_TOPICS,

random_state=RANDOM_STATE,

passes=PASSES,

alpha="auto",

per_word_topics=True

)

print("\nLDA model estimation completed.")

\# ============================================================

\# 9. Coherence calculation

\# ============================================================

coherence_model = CoherenceModel(

model=lda_model,

texts=texts,

dictionary=dictionary,

coherence="c_v",

processes=1

)

coherence_score = coherence_model.get_coherence()

print("c_v coherence:", round(coherence_score, 4))

\# ============================================================

\# 10. Topic keywords

\# ============================================================

topic_rows = []

for topic_id in range(NUM_TOPICS):

topic_terms = lda_model.show_topic(topic_id, topn=15)

print(f"\nTopic {topic_id + 1}")

print(topic_terms)

for rank, (word, probability) in enumerate(topic_terms, start=1):

topic_rows.append({

"topic_zero_based": topic_id,

"topic_reporting": topic_id + 1,

"rank": rank,

"word": word,

"probability": probability

})

topic_keywords = pd.DataFrame(topic_rows)

topic_keywords.to_excel(

OUTPUT_DIR / "lda_topic_keywords_top15.xlsx",

index=False

)

\# ============================================================

\# 11. Dominant topic assignment

\# ============================================================

def get_dominant_topic(ldamodel, corpus):

dominant_topics = []

dominant_probabilities = []

for document in corpus:

topic_distribution = ldamodel.get_document_topics(

document,

minimum_probability=0.0

)

topic_distribution = sorted(

topic_distribution,

key=lambda x: x[1],

reverse=True

)

dominant_topics.append(topic_distribution[0][0])

dominant_probabilities.append(topic_distribution[0][1])

return dominant_topics, dominant_probabilities

df["dominant_topic_zero_based"], df["dominant_topic_probability"] = (

get_dominant_topic(lda_model, corpus)

)

df["dominant_topic_reporting"] = df["dominant_topic_zero_based"] + 1

\# ============================================================

\# 12. Topic prevalence

\# ============================================================

topic_prevalence = (

df["dominant_topic_reporting"]

.value_counts()

.sort_index()

.reset_index()

)

topic_prevalence.columns = ["topic", "n"]

topic_prevalence["percent"] = (

topic_prevalence["n"] / topic_prevalence["n"].sum() * 100

)

print("\nTopic prevalence")

print(topic_prevalence)

topic_prevalence.to_excel(

OUTPUT_DIR / "lda_topic_prevalence.xlsx",

index=False

)

\# ============================================================

\# 13. Monthly topic distribution

\# ============================================================

monthly_topic_counts = (

df.groupby(["year_month", "dominant_topic_reporting"])

.size()

.unstack(fill_value=0)

)

monthly_topic_counts.to_excel(

OUTPUT_DIR / "monthly_topic_counts.xlsx"

)

plt.figure(figsize=(14, 6))

monthly_topic_counts.plot(kind="line", figsize=(14, 6))

plt.title("Monthly distribution of dominant topics")

plt.xlabel("Month")

plt.ylabel("Number of complaints")

plt.xticks(rotation=45)

plt.tight_layout()

plt.savefig(

OUTPUT_DIR / "monthly_topic_distribution.png",

dpi=300

)

plt.close()

\# ============================================================

\# 14. Save final analytical dataset

\# ============================================================

df.to_excel(

OUTPUT_DIR / "complaints_with_dominant_topics.xlsx",

index=False

)

lda_model.save(str(OUTPUT_DIR / "lda_model_13_topics.gensim"))

dictionary.save(str(OUTPUT_DIR / "gensim_dictionary.gensim"))

print("\nOutputs saved in:")

print(OUTPUT_DIR)
